# Local Pattern Transformation Based Feature Extraction for Recognition of Parkinson’s Disease Based on Gait Signals

**DOI:** 10.3390/diagnostics11081395

**Published:** 2021-08-01

**Authors:** S. Jeba Priya, Arockia Jansi Rani, M. S. P. Subathra, Mazin Abed Mohammed, Robertas Damaševičius, Neha Ubendran

**Affiliations:** 1 Department of Computer Science and Engineering, Manonmaniam Sundaranar University, Tirunelveli 627012, Tamil Nadu, India; Jebaleeban@gmail.com (S.J.P.); jansi_cse@msuniv.ac.in (A.J.R.); 2 Department of Computer Science and Engineering, Karunya Institute of Technology and Sciences, Coimbatore 641114, Tamil Nadu, India; nehaubendran61@gmail.com; 3 Department of Electrical and Electronics Engineering, Karunya Institute of Technology and Sciences, Coimbatore 641114, Tamil Nadu, India; subathra@karunya.edu; 4 Information Systems Department, College of Computer Science and Information Technology, University of Anbar, Ramadi 31000, Anbar, Iraq; mazinalshujeary@uoanbar.edu.iq; 5 Department of Applied Informatics, Vytautas Magnus University, 44404 Kaunas, Lithuania; 6 Faculty of Applied Mathematics, Silesian University of Technology, 44-100 Gliwice, Poland

**Keywords:** Parkinson’s disease, Parkinson’s gait, symmetrically weighted local neighbour gradient pattern, local pattern transformation, feature extraction

## Abstract

Parkinson’s disease (PD) is a neuro-degenerative disorder primarily triggered due to the deterioration of dopamine-producing neurons in the substantia nigra of the human brain. The early detection of Parkinson’s disease can assist in preventing deteriorating health. This paper analyzes human gait signals using Local Binary Pattern (LBP) techniques during feature extraction before classification. Supplementary to the LBP techniques, Local Gradient Pattern (LGP), Local Neighbour Descriptive Pattern (LNDP), and Local Neighbour Gradient Pattern (LNGP) were utilized to extract features from gait signals. The statistical features were derived and analyzed, and the statistical Kruskal–Wallis test was carried out for the selection of an optimal feature set. The classification was then carried out by an Artificial Neural Network (ANN) for the identified feature set. The proposed Symmetrically Weighted Local Neighbour Gradient Pattern (SWLNGP) method achieves a better performance, with 96.28% accuracy, 96.57% sensitivity, and 95.94% specificity. This study suggests that SWLNGP could be an effective feature extraction technique for the recognition of Parkinsonian gait.

## 1. Introduction

Parkinson’s disease (PD) is a neurological condition located in the basal ganglia and brainstem due to a lack of dopaminergic activity [1]. It is a chronic disorder of adult onset, which becomes more common with age [2]. A survey says that from 5% to 10% of cases are due to hereditary predisposition, and 90–95% of cases are due to idiopathic behavior [3]. Tremor, rigidity, and slowness of movement are the symptoms of PD in its early stage, causing difficulty in movements [4] and dysphonia, also known as speech disorder [5]. Sleep disorder, depression, and loss of smell are the symptoms that start before the commencement of physical disorders [6].

PD motor disorders are diagnosed using freezing of gait [7], foot pressure analysis, finger motion analysis, voice and speech disorders [8,9], brain dopaminergic imaging, and handwriting studies. However, the decision is made based on subjective feelings and the time taken for testing is extensive [10]. Chiu et al., on the other hand, state that finger motion analysis was used to test the goniometry of finger joints [11]. Based on the survey, the dopaminergic image in the brain is considered as an authentic method for the identification of PD [12].

In gait dynamics, continuous effects of PD were noticed. Gait idiosyncrasies involve the subject’s stride duration, pace, and swing period, which can easily be assessed by wearable sensors [13]. Richards et al. [14] reported on surveys of motor control signs in patients for the inference of a Unified Parkinson’s Disease Rating Scale (UPDRS) score to assess the severity of PD symptoms. These findings can still be misleading, despite their being checked by expert physicians. Chemical tests have been suggested to identify PD, which has been analyzed in [15]. Functional brain changes in magnetic resonance images (MRI) can be detected in people with PD [16] and Alzheimer’s disease [17]. Handwriting irregularities [18], and handwritten drawing patterns [19], especially the spiral drawing test, have been used to identify PD. Resting state or motor activation electroencephalography (EEG) analysis also have been found to be useful for neurodegenerative disorder classification [20,21].

The connection between gait and dynamic equilibrium subjects in the identification of PD in its early stage has been diagnosed using various advanced diagnostic software systems, which were proposed in [22]. In the traditional method of PD diagnosis, the gait signals are usually observed by trained neurologists to identify the neurological disorder, which is time-consuming and complex. Using information obtained from wearable sensors, the medical doctor can remotely assess the PD patient’s status and prescribe the best treatment [23]. Recently, mobile devices such as smartphones and tablets have been employed to analyze user input in the form of voice, spiral drawings and answers to the self-administered cognitive test (SAGE) [24] to detect the symptoms of PD and related central nervous system (CNS) disorders, such as Alzheimer’s disease, Huntington’s disease (HD), or mild cognitive impairment (MCI) [25,26].

In Local Binary Pattern (LBP) techniques, the time taken for the process of feature extraction is very minimal and each sample of the pattern can be analyzed. The development of pattern recognition techniques to select the prominent statistical features from gait signals with improved accuracy remains a challenging task in the automatic diagnosis of PD. Ertugrul et al. [4] presented a method for detecting PD using shifted one-dimensional LBPs by extracting the features from sensor readings and the possible shift amount was carried along with several machine learning algorithms to achieve the highest accuracy. The author of [27] developed a technique called neighborhood representation local binary patterns, in which the statistical features were extracted from the transformed data and the performance was evaluated using artificial neural network (ANN) classifier. Various methods of signal processing techniques are used in PD detection. Using the time domain analysis, methods such as Principal Component Analysis (PCA) [28], Neural Network with Weighted Fuzzy Membership function [29], and Short-Term Fourier Transforms (STFT) [30] are used to classify the Parkinson’s and non-Parkinson’s gait signals. PD is often not diagnosed for several years, because symptoms and the course of the disease differ. Accordingly, more sensitive diagnostic tools for the diagnosis of PD are required. Since the progression of the disease increases day by day, there is an increase in various symptoms that make PD more difficult to manage. The global burden of PD is increased with the increasing number of aged people. It is estimated that only 4% of people are diagnosed before the age of 50. It is evident that early prediction substantially reduces the risk of severity in PD patients through physical exercises. In the USA, it is reported that the average cost of Parkinson’s medication is $2,500 per year and Parkinson’s surgery costs up to $100,000 per patient. The proposed system will lead to the early diagnosis of the disease, which will reduce the average cost of medication for Parkinson’s patients.

The objective of this research is to determine and verify an intelligent system for detecting Parkinson’s disease from human gait signal. The dataset used in this paper was obtained from PhysioNet website, which provides movement disorder data supervised by a neurologist. PhysioNet is an open-source software that provides access to physiological and clinical data developed by the collaborative research of Harvard medical school, Boston University, McGill University and MIT. The gait signal in Parkinson’s disease (gaitpdb) dataset comprises three gait datasets recorded through various experimental setup, which have been used to identify Parkinson’s disease. This paper focuses on the role of pattern recognition approaches, namely LBP, Local Gradient Pattern, Local Neighbour Gradient Pattern and, Local Neighbour Descriptive Pattern, to detect PD.

The main contributions of our study are summarized as follows:

- This study investigates the role of analyzing gait signals using LBP techniques during feature extraction before classification. Supplementary to LBP techniques, Local Gradient Pattern (LGP), Local Neighbour Descriptive Pattern (LNDP), and Local Neighbour Gradient Pattern (LNGP) techniques are used to extract features from gait signals.
- Statistical features were derived and analyzed, and the statistical Kruskal–Wallis test was carried out for the selection of an optimal feature set. The classification was then carried out by ANN for the identified feature set.
- The highest performance accuracy was acquired by the Symmetrically Weighted Local Neighbour Gradient Pattern (SWLNGP) technique. The enhancement has been accomplished by the ability of local pattern techniques to gauge the gradient relationship between the neighboring points.
- To the best of the authors’ knowledge and from the literature survey, SWLNGP has not been used in PD detection to date.

Section 2 deals with a detailed discussion of the proposed methodology, including an explanation of the dataset. Section 3 explains the experimental results and discusses the comparison with other pattern techniques. Finally, Section 4 concludes the proposed study and offers future directions.

## 2. Materials and Methods

### 2.1. Dataset

The dataset (gaitpdb) utilized in this work was retrieved from PhysioNet, which was assembled by the authors of [31,32,33]. Gaitpdb encloses the gait signals that can lead to classifying the subject as healthy or Parkinson’s affected. Out of 166 subjects, 73 were healthy (mean age: 66.3 and mean weight: 72.4) and the rest were affected (mean age: 63.6 and mean weight: 72.8). The assimilated gait data were sampled at 0.01 s time intervals, for an epoch of two minutes. Inconsistencies were validated by requesting the subjects to accomplish three miscellaneous tasks. Based on the task executed by a subject and the progression of the dataset, they are entitled to labels such as Si [31], Ga [32], or Ju [33]. Sixteen sensors, eight on each foot, were integrated into shoes for supervision. The sensor signals collected from each foot channeled the Vertical Reaction Force (VRF), measured in Newton.

**Figure 1:**
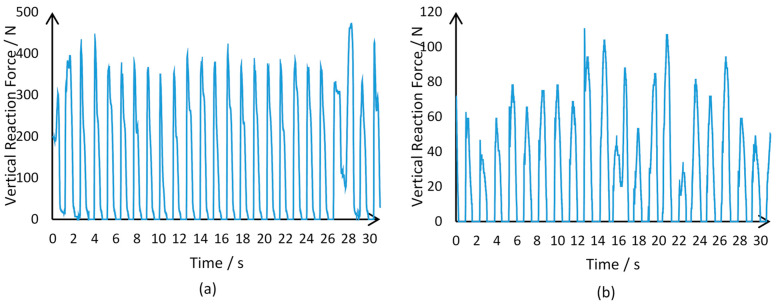
(**a**) illustrates VRF data captured by the sensors placed on the left foot of a subject declared healthy in the dataset; (**b**) illustrates VRF data captured by the sensor when placed in the same location on a Parkinson’s patient.

The experiments were performed on an Intel core i5 (2.40 GHz) processor with 6 GB RAM using the MATLAB R2018b (MathWorks, Nattick, MA, USA) software. The sampled data structure is disclosed in Table 1. For both Parkinson’s and healthy subjects, Figure 1 delineates the gait signals received.

In both Equations (1) and (2), n represents the length of the boolean array b.

### 2.2. Outline of Methodology

**Figure 2:**
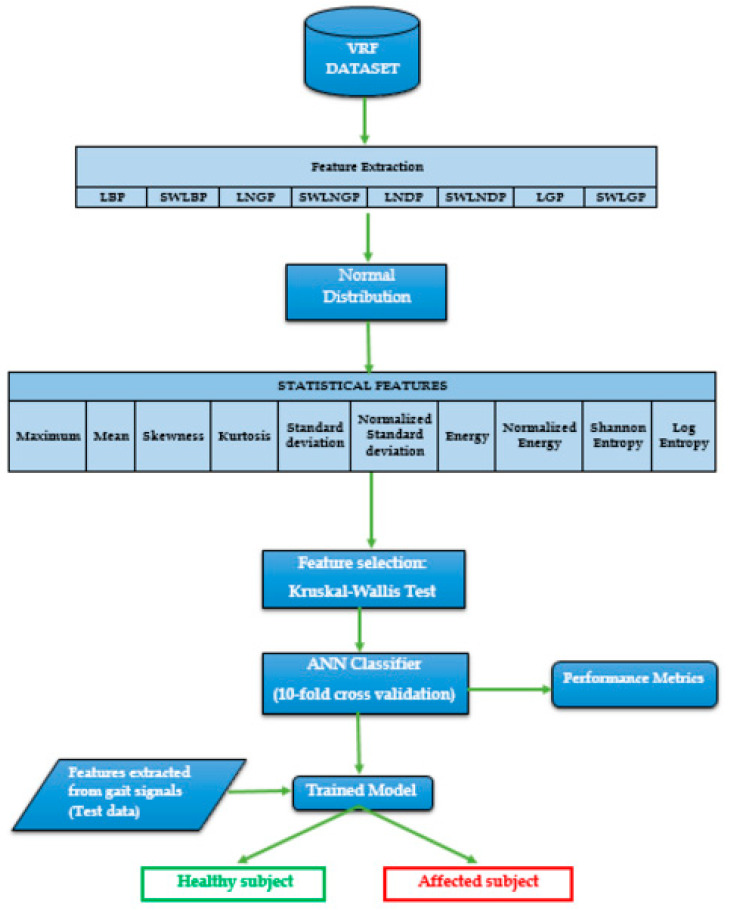
Block diagram of the proposed methodology.

Figure 2 illustrates an overview of the proposed methodology, followed by explanations regarding each block involved. The following section elaborations the rest of the process.

Block 1: Sensors positioned on the foot of the subject channels for calculating vertical ground reaction force (VRF) at the rate of 10 Hz for a time window of 120 s. The channeled signal data were stored in a numerical format for all 166 subjects in the dataset.

Block 2: Pattern recognition techniques process nine VRF samples to form a code representing the pattern, and the entire signal is converted into a normal distribution of values.

Block 3: The distribution’s statistical properties such as skewness, kurtosis, standard deviation, energy, normalized energy, Shannon’s entropy, log energy, entropy, mean, maximum, and normalized standard deviation were analyzed.

Block 4: Ten different features were evaluated from the normal distribution derived per sensor and, to enhance the model, the Kruskal–Wallis test was conducted to select the features with the highest impact on the categorization of the subjects.

Block 5: The extracted features are fed as an input to the artificial neural network classifier to distinguish between an affected patient and a healthy subject.

### 2.3. Feature Extraction

PD is commonly found amongst the elderly and its complications can be serious. Early detection of the disease may slow down the progression of the disease and decrease its fatality. Gait disturbance is a prominent symptom and occurs during the early stage of PD. Since traditional feature extraction techniques result in relatively poor classification accuracy, there is a need for a novel technique to identify the natural changes in the gait features for the early identification of PD, with improved accuracy. Based on this, a Symmetrically Weighted Local Neighbour Gradient Pattern (SWLNGP) method is proposed, and its performance is analyzed with the existing feature extraction algorithms. The foremost aim of this utilization is to select patterns from the images and support neural networks to boost its performance. The pattern identification procedure encompasses the transformation of a region of interest in the image into a decimal representation. At the end of this conversion, similar regions of the image hold identical decimal magnitude. By applying a pattern recognition algorithm, the signal is converted into a normal distribution with a fixed range. Furthermore, the distributions may be distinguished by extracting all the statistical features mentioned in Section 2.

In this study, various techniques were employed to analyze trends within VRF data sampled from the given subjects. Eight algorithms were implemented, and all are discussed in detail in the next section.

#### 2.3.1. Local Binary Pattern and Symmetrically Weighted Local Binary Patterns

Although eight algorithms were implemented, they are considered a derivative of LBP. LBP involves scrutinizing non-overlapping regions of the gait signal by comparing the eight values of VRF against a nominated value. A Boolean digit representing the result is concatenated to form a string of eight-bit length per comparison. The Boolean string is then transformed to decimal format. Through the repetitive processing of VRF data, a collection of unique values representing trends in gait signals are obtained. Although LBP is known for its ability to capture global variations, gait data have noise triggered due to random movements other than those requested.

**Figure 3:**
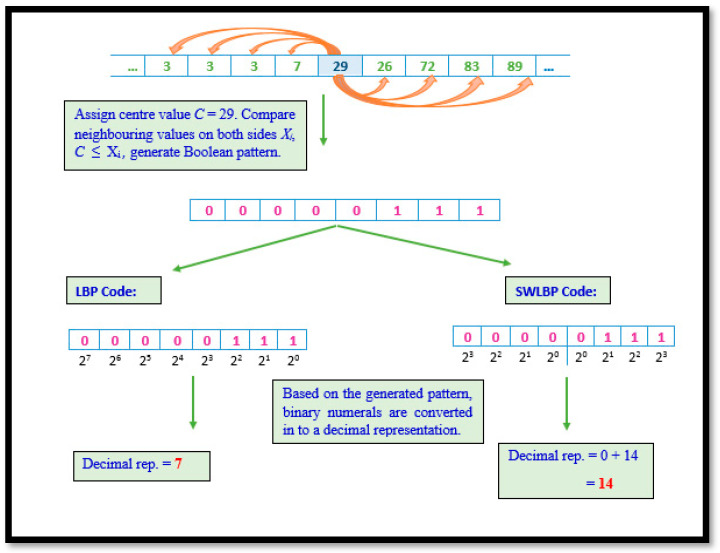
Illustration of LBP and SWLBP procedure.

Hence, a variation of the technique was put forward to mitigate the consequences of such noise. The difference between both techniques are represented in its binary to decimal conversion. Algorithm 1 elaborates the procedural representation of LBP and SWLBP pattern technique and Figure 3 depicts both LBP and SWLBP techniques.
**Algorithm 1:**Require: 1 × 9 VRF signal array, x[1…9].For a given signal, compute the centre point (c) with an adjacent point.For each sample point, calculate the gradient value asx[i]=|x[i]−c| for i=0,1,2,…,n.Compare the gradient values, if x[i] ≥ c, when the value of 1 is set, if not 0 will be assigned.Obtained binary code is converted into decimal representation.

The *LBP* and *SWLBP* code is calculated as:

(1) $$LBP=\sum_{i=1}^{n}\left(b\left[i\right]\times 2^{n-i}\right),$$

(2) SWLBP=∑i=1n2(b[i]×2n−i)+∑i=n2+1n(b[i]×2i−n2),

#### 2.3.2. Local Gradient Pattern and Symmetrically Weighted Local Gradient Pattern

Along with LBP’s procedural approach, LGP goes a step further to ensure that not only global variation, but also certain local dissimilarities in the signal, are captured. Hence, in this technique, the the mid-point value is selected and its VRF magnitudes are calculated based on their differences from the selected mid-points of the region. Furthermore, the derived values are averaged to form a threshold value. Unlike in LBP, the new derivations are compared to the threshold value instead of the mid-point, capturing major local variations as well.

**Figure 4:**
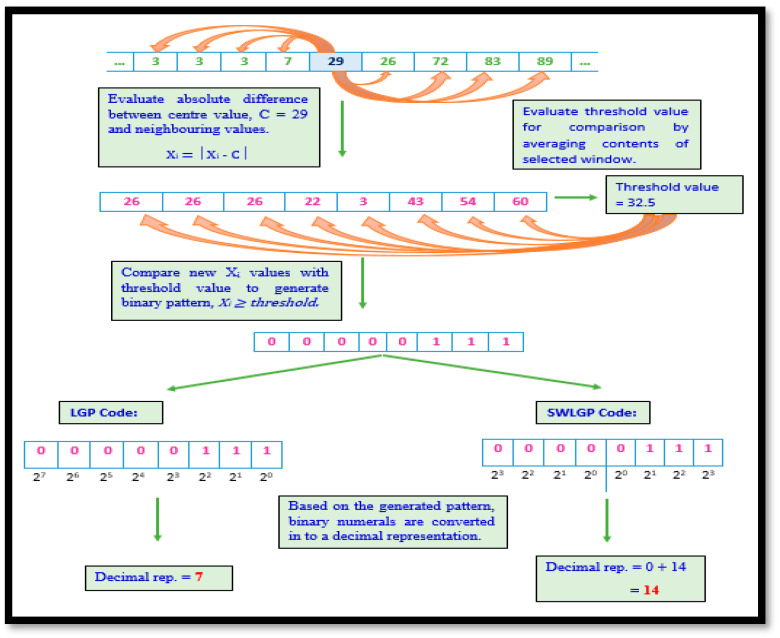
LGP procedure illustrated in a pictorial workflow diagram.

The SWLGP process follows all LGP steps, but disregards the traditional conversion method. Algorithm 2 elaborates the procedural representation of LGP and SWLGP pattern techniques and Figure 4 depicts both LGP and SWLGP techniques.
**Algorithm 2:**Require: 1 × 9 VRF signal array, x[1…9].Set center value in the region as c, with the number of adjacent points n.Evaluate the threshold value by implementing (3), and consider it the new comparison constant, since further comparisons are performed between the threshold value and the difference between the magnitude and value of c.Compute the absolute difference, the gradient g, between x[i] and c, where g=|x[i]−c|.Compare the gradient and the threshold value, g ≥ threshold and, if the result is true, represent by 1, if not indicate as 0.The resultant binary code is converted into a decimal code.

(3) $$Threshold=\frac{1}{n}\times \sum_{i=1}^{n}\left|x\left[i\right]-c\right|,$$

Mathematically, the *LGP* and *SWLGP* code is given as

(4) $$LGP=\sum_{i=1}^{n}\left(b\left[i\right]\times 2^{n-i}\right),$$

However, the variation in conversion illustrated by *SWLBP* is depicted as

(5) SWLGP=∑i=1n2(b[i]×2n−i)+∑i=n2+1n(b[i]×2i−n2),

#### 2.3.3. Local Neighbour Descriptive Pattern and Symmetrically Weighted Local Neighbour Descriptive Pattern

Unlike LBP and LGP, the LNDP methodology focuses on showing the prominence of local variation in gait signals. Therefore, this technique examines the relationship between a selected VRF magnitude within the region and its adjacent on the right. Such scrutiny warrants that any minute changes in trends within the signal are noticed and recorded. Though certain noise in gait data may reduce the efficiency of the technique in detecting intimate patterns, the utilization of SWLNDP helps the traditional method overcome this drawback. In SWLNDP or any other symmetrically weighted binary pattern technique, the conversion only yields values within the range 0–30, unlike in LBP, LGP or LNDP, where codes lay within the range 0–255.

**Figure 5:**
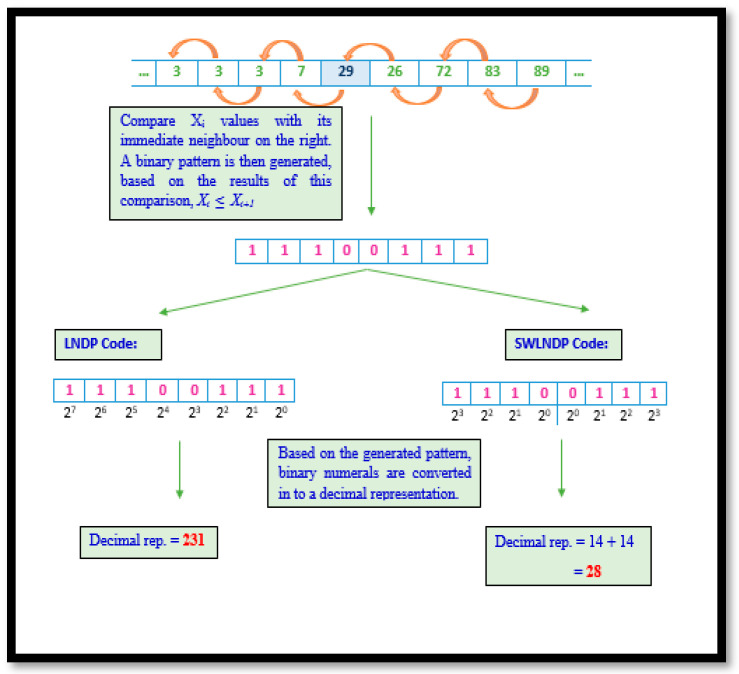
LNDP representation in a diagrammatical illustration.

The reduction in the limits of the range between the two sets of techniques symbolizes the difference in the ability to detect and uniquely present the features. Given the shrunken range in symmetrically weighted patterns, a set of trends are clustered into the same group, unlike in the traditional techniques. Therefore, reducing the uniqueness by a safe standard ensures that the noise in the gait signal is disregarded. Algorithm 3 elaborates the procedural representation of LNDP and SWLNDP pattern technique and Figure 5 depicts both LNDP and SWLNDP techniques.
**Algorithm 3:**Require: 1 × 9 VRF signal array, x[1…9].Let the number of adjacent points m.Select the m/2 number of neighbor points for each signal point c with respect to front and back.Calculate the difference between consecutive points.Comparison of the result is computed as, x[i] ≤ x[i+1], where 1 in case of current element greater than or equal to the adjacent value, else 0.In the encrypting step, the binary value is converted into a decimal value.
Mathematically, the LNDP and SWLNDP code is given as:

(6) $$LNDP=\sum_{i=1}^{m}\left(b\left[i\right]\times 2^{n-i}\right),$$

However, the variation in conversion illustrated by SWLNDP is depicted as

(7) SWLNDP=∑i=1n2(b[i]×2n−i)+∑i=n2+1n(b[i]×2i−n2),

#### 2.3.4. Local Neighbour Gradient Pattern and Symmetrically Weighted Local Neighbour Gradient Pattern

**Figure 6:**
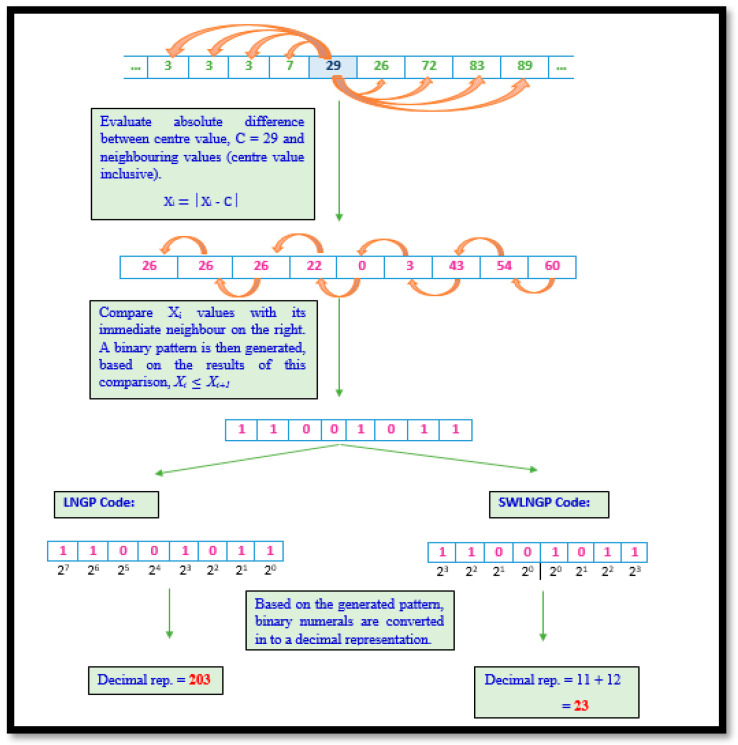
LNGP and SWLNGP algorithm illustration.

The LNGP technique is derived from LBP. The illustration demonstrated in Figure 6 associates phases from both LGP and LNDP. On combining these implementations, the LNGP technique sensitizes any minute local variations within the region being processed, in turn, capturing minor as well as major global variations in the signal being processed. Though this may be considered an advantage from certain perspectives, this may be a hindrance when extracting statistical features to determine the disparities between the records of healthy subjects versus the affected. Hence, the inclusion of SWLNGP was considered a necessity, as in all previously discussed algorithms. Thus, SWLNGP reduces the sensitivity of the algorithm and ensures that only vital changes in the signal data are captured. This contributes towards the efficient distinguishability of a distribution of values produced by gait signals of a healthy subject versus the distribution of values of an affected subject. The illustrations of LNGP and SWLNGP code are shown in Figure 6. Algorithm 4 illstrates the steps involved in computing LNGP and SWLNGP technique.
**Algorithm 4:**Require: 1 × 9 VRF signal array, x[1…9].Select the center value c from the set of neighboring points.For each sample point, calculate the gradient value as x[i]=|x[i]−c| for i=0,1,2,…,n.Compare the continuous neighboring gradient point along with the center value c. If the adjacent point x[i] is greater than or equal to x[i+1] the value of 1 is set, otherwise 0 will be assigned.In the transformation step, the binary value is converted into decimal code.Compute the *LNGP* code.

(8) $$LNGP=\sum_{i=1}^{n}\left(b\left[i\right]\times 2^{n-i}\right),$$

Hence, the mathematical formulation for SWLNGP was depicted as:

(9) SWLNGP=∑i=1n2(b[i]×2n−i)+∑i=n2+1n(b[i]×2i−n2),

Eleven features based on the transformation were extracted from each sensor and they are maximum, mean, standard deviation (*SD*), energy, skewness, and kurtosis, normalized energy (*NE*), normalized standard deviation (*NSD*), log entropy (*LE*), and Shannon’s entropy (*SE*), which are defined as follows:

(10) $$Mean\;\bar{c}=\frac{1}{N}\sum_{i=1}^{N}c_{i},$$

(11) $$SD\;\sigma \left(c\right)=\sqrt[{}]{\frac{1}{N}\sum_{i=1}^{N}{\left(c_{i}-\bar{c}\right)}^{2}},$$

(12) $$Energy\;e=\sum_{i=1}^{M}{\left(c_{i}\right)}^{2},$$

(13) Skewness s=N(N−1)(N−2)∑i=1N(ci−c¯σ(c))3,

(14) $$Kurtosis\;k=\frac{1}{N\times {\left(\sigma \left(c\right)\right)}^{4}}\sum_{i=1}^{N}{\left(c_{i}-\bar{c}\right)}^{4},$$

(15) $$Normalized\;Energy\;NE=\frac{1}{N}\sum_{i=1}^{N}{\left(c_{i}\right)}^{2},$$

(16) $$Normalized\;standard\;deviation\;NSD=\frac{\sigma \left(c\right)}{c_{max}-c_{min}},$$

(17) $$Log\;Entropy\;LE=\sum_{i=1}^{N}log_{2}{\left(c_{i}\right)}^{2},\;$$

(18) $$Shannon\;Entropy\;SE=-\sum_{i=1}^{N}{\left(c_{i}\right)}^{2}log_{2}\left(c_{i}{}^{2}\right).$$

In the above-stated equations, the variable $c_{i}$ denotes the decimal code at position i in the distribution of values and, N denotes the number of codes that are available.

### 2.4. Feature Selection

Feature selection helps to diminish the computational complexity in classifying PD and normal patients. Some of the extracted features, due to their low discriminating abilities, do not contribute significantly to the results. Moreover, not all features are very much in line with the result. In this study, the non-parametric Kruskal-Wallis test was conducted to figure out the significance of each feature for identifying the difference between the PD and healthy subjects. This test can be used to analyze statistical differences between two or more features of an independent variable.

To compute the Kruskal–Wallis test statistic K, where n represents the number of features, J refers to the total of samples, $A_{j}$ represents the sample size in j-th group and $T_{j}$ denotes the ranking function, the following equation was used:

(19) $$K=12/J\left(J+1\right)=\sum_{j=1}^{n}\left(\frac{Tj^{2}}{Aj}\right)-3\left(J+1\right),$$

The statistical features were considered as a set of independent features and targets were considered as the dependent feature. A p-score is originated by the test, which portrays the impact on the target. The feature is checked as impactful if its p-score is lower than the threshold value of 0.05. The extracted features are summarized in Table 2. The results obtained stress that the lowest p-scores were reached by skewness, kurtosis, and normalized standard deviation.

### 2.5. Artificial Neural Network (ANN) Classifier

**Figure 7:**
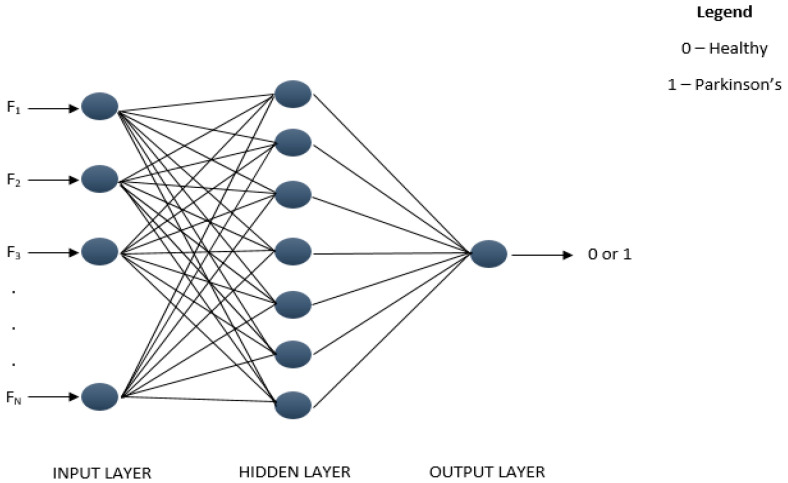
Artificial Neural Network Architecture.

An ANN classifier is a group of neurons performing a mathematical operation on each layer, as shown by Figure 7. The neural network architecture involved in this work is shown in Table 3, demonstrating the training feature that yielded the best results. The architecture can be sorted into an ANN classifier by performing various training functions, changing the hidden layer and other parameters.

However, only the training role was examined to evaluate the difference in output in this analysis. The nine different training functions applied in this work are Scaled Conjugate Gradient (trainscg), Conjugate Gradient with Powell/Beale Restarts (traincgb), Fletcher-Powell Conjugate Gradient (traincgf), Polak-Ribiére Conjugate Gradient (traincgp), One Step Secant (trainoss), Gradient Descent (traingd), Variable Learning Rate Gradient Descent (traingdx), Gradient Descent with Momentum (traingdm), and Resilient Backpropagation (trainrp).

The performance of the classifier was discerned using Positive (*TP*), True Negative (*TN*), False Positive (*FP*), and False Negative (*FN*) parameters, as follows:

(20) $$Accuracy=\frac{TN+TP}{TN+TP+FN+FP}\times 100,$$

(21) $$Sensitivity=\frac{TP}{TP+FN}\times 100,$$

(22) $$Specificity=\frac{TN}{TN+FP}\times 100,$$

(23) $$PPV=\frac{TP}{TP+FP}\times 100,$$

(24) $$NPV=\frac{TN}{TN+FN}\times 100,$$

(25) $$MCC=\frac{\left(TP\times TN\right)-\left(FP\times FN\right)}{\sqrt{\left(TP+FP\right)\left(TP+FN\right)\left(TN+FP\right)\left(TN+FN\right)}}\times 100,$$

(26) $$F1-Score=\frac{2\times TP}{FP+FN+\left(2\times TP\right)}\times 100,$$

(27) $$G-Mean=\sqrt{\left(\frac{TP}{TP+FN}\right)\times \left(\frac{TN}{TN+FP}\right)}\times 100,$$

Here, *PPV* and *NPV* are Positive Predictive Value and Negative Predictive Value. Sensitivity indicates the rate of positive values correctly deduced, while specificity determines the rate of negative values that were correctly distinguished. Accuracy measures the percentage of all correctly recognized values.

## 3. Results and Discussion

The prime objective of this study was to observe the influence of binary pattern techniques when identifying affected subjects from healthy persons using gait signals. Eight different pattern recognition techniques were used for the feature extraction, which leads to a good performance in identifying PD in its early stage. Divergence in the steps followed by each algorithm brings about different sets of dissimilarities within the trends in the VRF data. The proposed methodology, which contributes to maximum performance with respect to time-efficiency, classification was performed utilizing the statistical features derived from the transformed signal. In this phase of work, the resulting feature set per algorithm was classified where each set consists of 11 features per sensor, with a total of 176 features represented in the columns.

The classification was conducted twice in this work, under different conditions. On the first conditional event, the ANN classified all eight derived feature sets separately. The results of these proceedings are summarized in Table 4. However, based on the fundamentals of machine-learning techniques, it is well known that not all independent features contribute to classification. To overcome this drawback, a feature selection phase, as discussed in the previous section, was implemented. On scrutinizing the outcomes of the Kruskal–Wallis test, the p-score achieved by statistical features revealed that skewness, kurtosis and normalized standard deviation were the lowest. In theory, a lower the p-score for an independent feature leads to a higher correlation.

After these results, a revised feature set is formed for every algorithm attained; only skewness, kurtosis, and normalized standard deviation were derived for all sensors by reducing the number of independent variables to 48. Furthermore, the same ANN then classified the revised datasets per algorithm, and these results are summarized in Table 5. On scrutinizing the results from both classification phases, the performance of the ANN on the revised feature set, corresponding to SWLNGP, showed the maximum. With further research, it was found that the varying training functions of a neural network may significantly impact its performance. To explore the results of the proposed model, different training functions were configured, and a classification was performed.

Nine training functions were considered in this study, and Table 6 summarizes the results of classification under each configuration. Upon scrutinizing the performance reports, scaled conjugate gradient training function achieved a maximum accuracy of 96.28%, 96.57% sensitivity, and 95.94% specificity, when compared to the other training functions.

**Figure 8:**
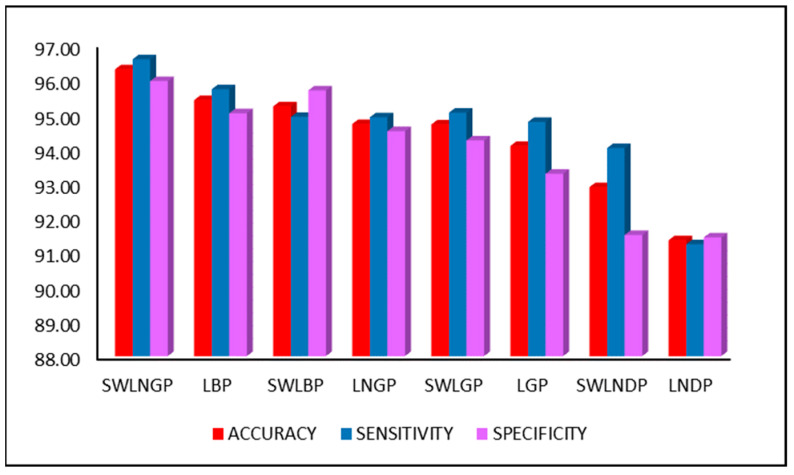
Comparison between performance metrics achieved by other techniques.

Figure 8 compares the three major performance metrics, accuracy, sensitivity, and specificity, during the second classification phase, involving the revised feature set. However, the primary reason for the rapid increase in performance is the SWLNGP algorithm. This algorithm can identify both local and global variation within the gait signal, which balances out the effects of noise. Therefore, the algorithm contributes heavily towards the achievement of an accuracy of this magnitude.

In comparison to other methods of feature extraction and machine-learning models proposed by existing authors, the technique suggested in this study is considerably more time-efficient. For example, Lee et al. [29] suggested using Wavelet Transforms and Fourier Transforms for feature extraction. Although these algorithms are widely used for signal processing, the mathematical operations involved in this algorithm make them more complex and, in terms of processing, a huge dataset leads to time complexity. Furthermore, the models suggested in [30,34] had only 74.32% and 89.92% accuracy, respectively.

Jane et al. [34] suggested the use of a Q-backpropagated time-delay neural network with no feature extraction. Though the model had an acceptable accuracy of 91.53%, the training of a complicated neural network may take more time when fed to a large number of observations. Perumal et al. [3] recommended the use of Analysis of Variance (ANOVA) tests combined with LDA, but this achieved an accuracy that peaked at 86.9%. Although comparatively time-efficient, the performance was still lower. Ertrugul et al. [4] suggested a pattern technique for the analysis of gait data; the research article focused on identifying the optimum position for comparison. By combining extracted features with a Multi-Layer Perceptron (MLP) classifier, the model achieved an accuracy of 88.89%.

**Figure 9:**
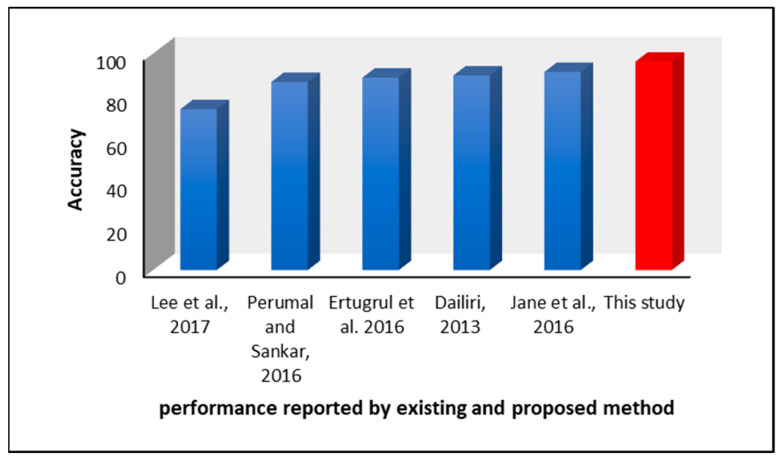
Comparison of performance reported by existing studies versus the proposed method.

However, the model proposed in this research has overtaken these reported performances by a valid margin. Figure 9 shows a diagrammatical representation of the performance metrics achieved by the discussed studies. In addition to canceling noise during conversion, not only SWLNGP but all other pattern techniques were proved to be time-efficient. In theory, these procedures do not involve any complex equations or evaluations. The most complex operation performed during the execution of such a technique is raising two to a certain power. As the data are processed nine cells at a time, the maximum power is be raised to seven. As proof of this theoretical derivation, the average time taken by each technique to analyze a single sensor input and process an entire patient record is summarized in Table 7.

Examining the values presented in this work proves that the techniques are highly time-efficient in converting signals and are almost instantaneous. This proves to be an advantage, as the patient is not asked to wait for hours or days to obtain their results. However, a neural network, depending on the complexity of the data provided, may take days to train. Each time a classification is carried out on deployment, the model may require training on the new dataset to include the latest prediction results. If the neural network requires days or hours to train, it may hinder the process of testing for a considerable amount of time. However, due to the convergence of gait signals and extraction of certain features, the new dataset is significantly less complex. The reduction in the complexity of the input ensures faster training times, as well as proof of this presumption: the classification times reported in this work are summarized in Table 8.

Examining the time values represented in Table 7, the time taken to run a 10-fold cross-validation, averaged for 10 runs, as well as the average time per single training and validation for each algorithm is only seconds. This ensures that there would be no time delay longer than a maximum of minutes before the model is ready for utilization if applied in real-life situations. Continuous training of the model with new and old data for each prediction means that the performance of the model may increase. A handful of reasons could be provided for the marginal decrease in performance, one of them being that the dataset consists of gait data from multiple tasks. The Ga set of subjects performed tasks varying from that of Si and Ju. The variation in tasks introduces different trends to the VRF, diminishing the pattern technique’s ability to capture similar trends.

Furthermore, the Ju subset of the subject records had an inconsistent number of samples, not the standard time samples for 120s. Hence, due to the lack of data in the Ju subset, the techniques may not identify patterns as efficiently as possible. A possible enhancement of this study could be the introduction of regression algorithms to predict the H & Y scale. The gaitpdb dataset utilized in this study had the H & Y score of each subject. By utilizing the revised dataset, finalized as the optimum feature set, regression algorithms may then be implemented to forecast the H & Y scale of a subject. This could also contribute to the indirect enhancement of the accuracy of the model. The confusion matrix for the maximum accuracy is given Figure 9.

The accuracy obtained in this study is greater than the results obtained by [3,4,29,30,34] and is represented in Figure 8 and Table 9.

**Figure 10:**
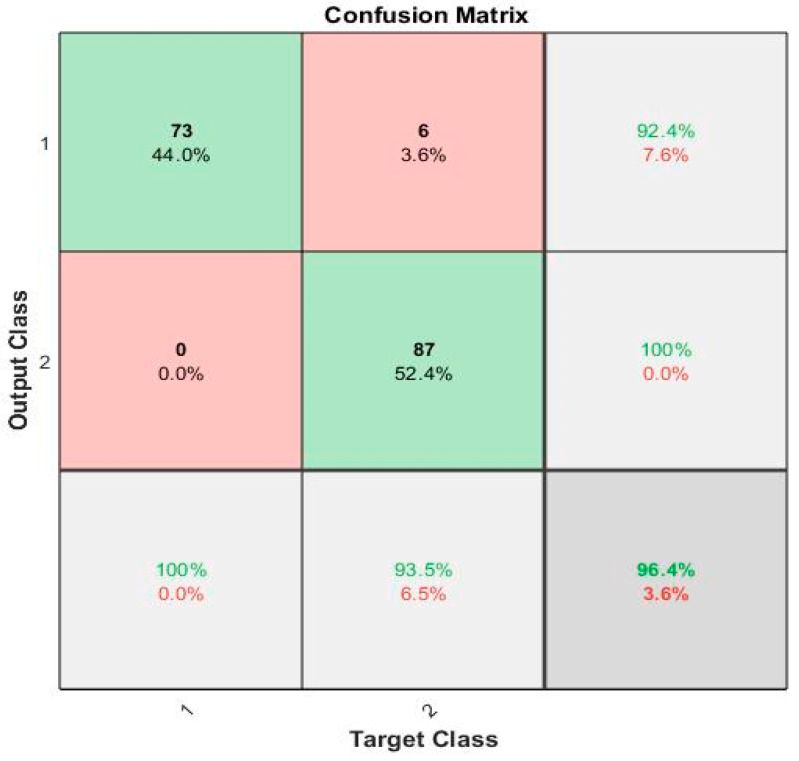
The confusion matrix for the maximum accuracy obtained by the classifier.

The confusion matrix for the proposed local pattern-transformation-based feature extraction technique for recognition of Parkinson’s gait signals is shown in Figure 10.

## 4. Conclusions

In this work, the classification of the PD patients was carried out using one-dimensional VRF sampled data, which were collected from 16 channels at regular time intervals. Eight different pattern recognition techniques were proposed for the diagnosis of Parkinson’s disease using gait signals. In the proposed algorithms, the gradient values were extracted from the signals as the statistical features for the classification of PD in its early stage. To differentiate between PD and control (healthy) patients, the extracted features were tested using the Kruskal–Wallis test to check the importance of each feature. By testing the identical distribution of every feature, a p-score was obtained. The features obtaining a p-value of less than 0.05 were considered statically significant and represent strong evidence against the null hypothesis. According to the result obtained from this analysis, a few features were selected for classification. The proposed algorithms were analyzed for 16 different channels and features using the ANN classifier, and the Stochastic Gradient training function was shown to be superior. The ANN classifier achieved a maximum accuracy of 96.28% for classifying PD and non-PD subjects using gait signals. In further analysis, these techniques were proven to be time-efficient, and revising the datasets significantly diminished the training time for the ANN. However, the inconsistency of samples and variations, caused due to a diversity of tasks, contributed towards the reduction in performance. To enhance the performance, it is possible to implement regression procedures to predict the H & Y scores, and the disease severity can also be diagnosed.

## Figures and Tables

**Table 1:** Columns and data represented in a patient’s record.

Columns	Data Represented
Col 1	Time (seconds)
Cols 2–9	Vertical Ground Reaction Force from sensors in left foot (Newton)
Cols 10–17	Vertical Ground Reaction Force from sensors in right foot (Newton)
Col 18	Total Reaction Force under left foot (Newton)
Col 19	Total Reaction Force under right foot (Newton)

**Table 2:** Results of the Kruskal–Wallis test for feature selection.

Feature	P-Score
Standard Deviation	0.081195
Energy	0.077301
Normalized Energy	0.077301
Shannon’s Entropy	0.072003
Log Energy Entropy	0.067493
Mean	0.033388
Maximum	0.005222
Skewness	2.00 × 10^−4^
Kurtosis	1.43 × 10^−4^
Normalized Standard Deviation	1.34 × 10^−4^

**Table 3:** ANN configuration summary and input size.

Parameters	Values
No. of Input Neurons	176/48 (Depending on input feature set)
No. of Hidden Neurons	20
No. of Output Neurons	1
Training Function	Trainscg
Hidden Transfer Function	Tansig
Output Transfer Function	Softmax

**Table 4:** Results of first classification phase: all feature columns fed as input to the ANN.

Techniques	Accuracy (%)	Sensitivity (%)	Specificity (%)	PPV (%)	NPV (%)	MCC (%)	F1-Score (%)	G-Mean (%)
LBP	95.08	95.70	94.26	95.80	94.86	90.31	95.57371	94.81898
SWLBP	95.50	95.23	95.80	96.84	94.48	91.17	95.88928	95.39281
LGP	94.43	93.98	94.96	96.04	93.14	89.06	94.84712	94.30776
SWLGP	93.83	93.66	94.03	95.45	92.44	87.79	94.37416	93.649
LNDP	92.38	92.86	91.69	93.93	91.15	84.811	93.18298	91.9793
SWLNDP	93.50	93.74	93.16	94.32	93.37	87.29	93.79522	93.23528
LNGP	94.42	94.42	94.46	95.75	94.10	89.33	94.78071	94.18657
SWLNGP	94.64	94.51	94.87	95.70	93.95	89.52	94.95906	94.59342

**Table 5:** Results of second classification phase: revised feature set input to the ANN.

Techniques	Accuracy (%)	Sensitivity (%)	Specificity (%)	PPV (%)	NPV (%)	MCC (%)	F1-Score (%)	G-Mean (%)
LBP	95.39	95.71	95.01	96.48	94.21	90.73	95.94554	94.87847
SWLBP	95.21	94.91	95.67	96.33	94.61	90.76	95.38315	95.06822
LGP	94.07	94.76	93.26	95.16	93.57	88.37	94.68777	93.76139
SWLGP	94.69	95.03	94.23	95.69	94.26	89.59	95.17081	94.38679
LNDP	91.34	91.23	91.42	93.09	90.34	83.04	91.85903	91.09237
SWLNDP	92.88	94.01	91.5	93.68	92.60	85.87	93.6776	92.5154
LNGP	94.70	94.90	94.5	95.48	94.17	89.52	95.0827	94.59604
SWLNGP	96.28	96.57	95.94	96.94	96.04	92.75	**96.6132**	**96.13201**

**Table 6:** Results from classification under different training functions.

Training Function	Accuracy (%)	Sensitivity (%)	Specificity (%)	PPV (%)	NPV (%)	MCC (%)	F1-Score (%)	G-Mean (%)
trainscg	96.28	96.57	95.94	96.94	96.04	92.75	**96.6132**	**96.13201**
traincgb	94.97	95.01	94.92	96.06	94.25	90.12	95.36571	94.8344
trainoss	94.48	95.15	93.66	95.17	93.96	88.96	95.07893	94.27387
traincgf	93.91	94.07	93.69	95.11	93.22	88.04	94.36148	93.66999
traincgp	92.83	93.43	92.07	94.02	92.06	85.77	93.57451	92.57581
trainrp	91.80	91.90	91.73	93.56	90.95	84.05	92.40578	91.52484
traingdx	81.46	83.11	79.28	83.99	80.34	63.32	83.00961	80.60864
traingd	66.46	70.26	61.51	70.64	63.50	32.87	-	63.52383
traingdm	63.6969	68.12	58.01	67.95	59.88	26.91	67.2902	61.52082

**Table 7:** Time taken to convert sensor data by pattern techniques.

	LBP	SWLBP	LGP	SWLGP	LNDP	SWLNDP	LNGP	SWLNGP
Average time taken to convert sensor data (s)	0.0060	0.0059	0.0084	0.0059	0.0054	0.0054	0.0054	0.0054
Average time taken per patient file (s)	0.0960	0.0942	0.1351	0.0936	0.0864	0.0872	0.0869	0.0863

**Table 8:** Time taken to classify both actual and revised dataset in seconds.

		LBP	SWLBP	LGP	SWLGP	LNDP	SWLNDP	LNGP	SWLNGP
All features	10-fold cross validation	5.66	4.70	5.69	5.34	4.89	4.91	5.53	5.46
	Per training and validation	0.57	0.47	0.57	0.53	0.49	0.49	0.55	0.55
Selected features	10-fold cross validation	3.79	3.65	3.55	3.63	3.72	3.44	3.21	3.09
	Per training and validation	0.38	0.36	0.35	0.36	0.37	0.34	0.32	0.31

**Table 9:** Comparison between performance metrics achieved by other pattern techniques.

Reference	Methods	Accuracy (%)	Sensitivity (%)	Specificity (%)
Lee et al. [29]	Wavelet transforms for feature extraction and classification by weighted fuzzy membership functions.	74.32	81.63	73.77
Dailiri [30]	Short-time Fourier Transforms were employed for feature extraction and reduced by chi-square method before classification by Support Vector Machines.	89.92	91.71	91.20
Jane et al. [34]	Time series data of a patient’s gait were directly fed to the model. The model comprises a Q-Back propagated time-delay Neural Network trained using a Q-learning back propagation algorithm.	91.53	-	-
Perumal and Sankar [3]	ANOVA test performed on VRF from the left foot. Various classifiers were implemented, and Linear Discriminant Analysis was chosen.	86.9	-	-
Ertugrul et al. [4]	1-D Shifted LBP was used in feature extraction and multiple classifiers were tested. Multilayer Perceptron was the highest performing classifier.	88.89	88.9	82.2
The proposed study	Features extracted using Symmetrically Weighted Local Neighbour Gradient Pattern were classified by ANN.	96.28	96.57	95.94

## Data Availability

The data is available from the Physionet database at https://physionet.org/content/gaitdb/1.0.0/, accessed on 17 June 2021.
